# Enhanced Recovery After Surgery for Patients Undergoing Cytoreductive Surgery and Hyperthermic Intraperitoneal Chemotherapy: A Systematic Review and Meta-Analysis

**DOI:** 10.3389/fsurg.2021.713171

**Published:** 2021-07-21

**Authors:** Feng Mao, Zhenmin Huang

**Affiliations:** ^1^Department of Thyroid/Vascular Surgery, Huzhou Cent Hospital, Affiliated Cent Hospital HuZhou University, Huzhou, China; ^2^Department of Galactophore/General Surgery, Huzhou Cent Hospital, Affiliated Cent Hospital HuZhou University, Huzhou, China

**Keywords:** ERAS, peritoneal neoplasm, abdominal malignancy, cytoreductive surgery, HIPEC

## Abstract

**Background:** Cytoreductive surgery (CRS) and hyperthermic intraperitoneal chemotherapy (HIPEC) is a promising approach for the management of peritoneal carcinomatosis, but is associated with significant morbidity and prolonged hospital stay. Herein, we review the impact of Enhanced recovery after surgery (ERAS) protocol on length of stay (LOS) and early complications in patients undergoing CRS and HIPEC for peritoneal carcinomatosis.

**Methods:** PubMed and Embase were searched for studies comparing ERAS protocol with control for CRS + HIPEC. Mean difference (MD) and risk ratios (RR) were calculated for LOS and complications respectively.

**Results:** Six retrospective studies were included. Meta-analysis indicated statistically significant reduction in LOS with ERAS (MD: −2.82 95% CI: −3.79, −1.85 I^2^ = 29% p < 0.00001). Our results demonstrated significantly reduced risk of Calvien Dindo grade III/IV complications with the use of ERAS protocol as compared to the control group (RR: 0.60 95% CI: 0.41, 0.87 I^2^ = 0% p = 0.007). Pooled analysis of limited studies demonstrated no statistically significant difference in the risk of reoperation (RR: 1.04 95% CI: 0.54, 2.03 I^2^ = 50% p = 0.90) readmission (RR: 0.55 95% CI: 0.21, 1.49 I^2^ = 0% p = 0.24), acute kidney injury (RR: 0.55 95% CI: 0.28, 1.10 I^2^ = 0% p = 0.09) or mortality (RR: 0.62 95% CI: 0.17, 2.26 I^2^ = 0% p = 0.46) between the study groups.

**Conclusion:** For CRS + HIPEC, ERAS is associated with significantly reduced LOS along with lower incidence of complications. Limited data suggest that use of ERAS protocol is not associated with increased readmission, reoperation, and mortality rates in these patients. There is a need for randomized controlled trials to corroborate the current evidence.

## Introduction

Peritoneal carcinomatoses are a heterogeneous group of disease which can either arise primarily from the peritoneum itself or metastasis of other tumors in the abdomen located at the colon, rectum, appendix, stomach or ovary (1). Indeed, the treatment plan varies with the disease histology and the extent of peritoneal involvement; but if left untreated, survival with this disease can be as low as 4 months (2).

Over the last two decades, cytoreductive surgery (CRS) and hyperthermic intraperitoneal chemotherapy (HIPEC) have emerged as an encouraging treatment option for managing peritoneal carcinomatosis (3, 4). While the surgical component of the regimen aims to eliminate the perceivable tumor mass via peritonectomy and visceral resections, the HIPEC component eradicates the microscopic disease (4). Studies have shown that in selected patients, the combination of CRS + HIPEC can lead to significant improvement in survival as compared to CRS or HIPEC alone (5, 6). Historically, CRS + HIPEC was known to cause a high incidence of morbidity and mortality owing to the significant physiological insult associated with the treatment (7, 8). However, recent data suggest that the safety of CRS + HIPEC is similar to that of other high-risk oncological procedures and increased morbidity with this treatment is a misperception from early experience (9). Nevertheless, this relatively resource-intensive therapy is offered by limited healthcare setups worldwide with varying perioperative practices which can significantly influence early patient outcomes (10).

Over the years, there has been an effort in the surgical community to improve patient care by following standard perioperative protocols (11). One such popular guideline is the enhanced recovery after surgery (ERAS) protocol which was initially developed for colorectal surgeries (12). ERAS is a multimodal strategy that incorporates many evidence-based preoperative, perioperative, and postoperative guidelines to improve patient recovery by reducing hospital stay and complication rates (13). However, despite the benefits offered by the ERAS protocol, concerns have also been raised regarding the increased risk of readmissions and acute kidney injury (AKI) due to the stringent fluid management guideline associated with the protocol (14, 15). While researchers have used some components of ERAS like pre-habilitation or restrictive fluid therapy for patients undergoing CRS + HIPEC (16, 17), the impact of a comprehensive ERAS protocol on these patients is still unclear. In the past 3 years, some researchers have presented their experience of ERAS with CRS + HIPEC but with a limited sample size (18, 19). To the best of our knowledge, no review has been attempted to synthesize data from these studies to present the best available evidence regarding the impact of ERAS on CRS + HIPEC. Therefore, the purpose of this study was to compare outcomes of patients undergoing CRS + HIPEC with and without the use of ERAS protocol.

## Materials and Methods

We performed this review in accordance with the recommendations of the PRISMA statement (Preferred Reporting Items for Systematic Reviews and Meta-analyses) (20). It is, however, clarified that the protocol was not preregistered on any online database.

### Study Selection

For clarity, we defined the Inclusion criteria based on the PICOS (Population, Intervention, Comparison, Outcome, and Study design) format as follows:

Population: Adult patients with peritoneal carcinomatosis undergoing CRS + HIPEC

Intervention: ERAS protocol

Comparison: Non-ERAS protocol (control)

Outcomes: Length of hospital stay (LOS) or complications

Study design: All types of studies

The following studies were excluded: (1) Studies without a control group. (2) Studies not reporting relevant outcome data (3) Studies only on CRS. (4) Review articles and non-English language studies. Where studies presented data from the same database with the same or overlapping study period, we included the study presenting larger sample size data.

### Search for Primary Studies

With the help of a librarian, we searched the databases of PubMed and Embase to look for relevant studies. The databases were screened from inception to 15th February 2021. Two reviewers independently conducted the electronic search with the following keywords: “enhanced recovery”, “ERAS”, “fast recovery”, “accelerated rehabilitation”, “multimodal perioperative care”, “cytoreductive surgery”, and “hyperthermic intraperitoneal chemotherapy”. Supplementary Table 1 demonstrates the search strategy. Every search result was evaluated by the two reviewers independently, initially by their titles and abstracts and then by full texts of relevant publications. All full-texts were reviewed based on the inclusion and exclusion criteria and the article satisfying all the criteria was finally selected for this review. Any disagreements were resolved by discussion. To avoid any missed studies, the bibliography of included studies was hand searched for any additional references.

### Data Extraction and Risk of Bias Assessment

We prepared a data extraction form at the beginning of the review to extract relevant details from the studies. The final version of this template was approved by all the study investigators. Details of the first author, publication year, study type, HIPEC drugs, sample size, demographic details, peritoneal cancer index (PCI), site of the primary tumor, operative time, ERAS protocol, and outcomes were extracted. Data were extracted by two reviewers independent of each other. Any disagreements were resolved by discussion. Outcomes assessed via a meta-analysis were, LOS, complications [grade III/IV based on Calvien Dindo classification (21)], readmission rates, reoperation rates, acute kidney injury (AKI), and mortality.

The methodological quality of included studies was assessed using the Newcastle-Ottawa scale (NOS) (22). This too was carried out in duplicate and independently by two study investigators. Studies were awarded points for selection of study population, comparability, and outcomes. The maximum score which can be awarded is nine.

### Statistical Analysis

The software “Review Manager” [RevMan, version 5.3; Nordic Cochrane Centre (Cochrane Collaboration), Copenhagen, Denmark; 2014] was used for the meta-analyses. As LOS is a continuous variable, we extracted mean and standard deviation (SD) data from the studies and pooled it to calculate the mean difference (MD) with 95% confidence intervals (CI). Median, range, or interquartile range data were converted into mean and SD when required using the method of Wan et al. (23). For the remaining categorical variables, we calculated risk ratios (RR) with 95% CI. Since there was already methodological heterogeneity in the included studies, we preferred a random-effects model for the analysis. The I2 statistic was used to assess inter-study heterogeneity. I2 values of 25–50% represented low, values of 50–75% medium, and >75% represented substantial heterogeneity. Funnel plots were used to assess publication bias (24).

## Results

**Figure 1:**
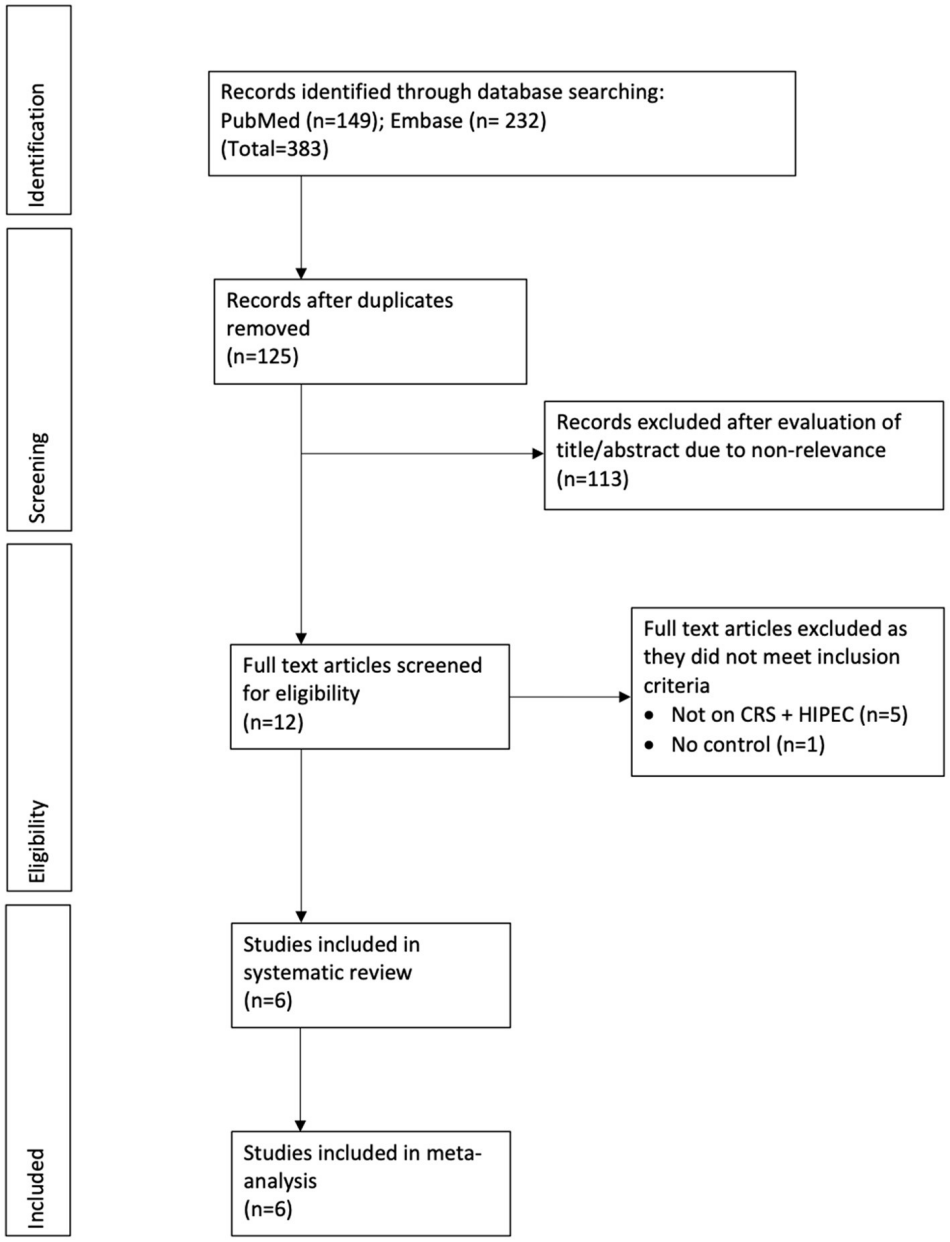
Study flow chart.

The flow-chart of the study is presented in Figure 1. The literature search revealed 383 records from both the databases combined. After deduplication, 125 unique records were screened of which 113 were excluded after title/abstract screening. From the remaining 12 articles, six were excluded after full-text analysis with reasons, and a total of six studies were included for this review (18, 19, 25–28).

Details of included studies are presented in Table 1. No randomized controlled trials (RCTs) were available and all were retrospective studies. Mitomycin, cisplatin, and oxaliplatin were used for the chemotherapy in the included studies. The sample size of the ERAS arm varied from 15 to 81 patients while in the control group it varied from 11 to 105 patients. The NOS score of the studies varied from 5 to 6. None of the studies carried out baseline matching of the study groups. A detailed description of the ERAS protocol in the pre-operative, intra-operative, and post-operative periods for each of the included studies is presented in Table 2.

**Table 1:** Details of included studies.

**References**	**Study type**	**Chemotherapy drug**	**Sample size**		**Male gender (%)**		**Mean age (years)**		**PCI**		**Site of origin**		**Operative time**		**NOS score**
			**ERAS**	**Control**	**ERAS**	**Control**	**ERAS**	**Control**	**ERAS**	**Control**	**ERAS**	**Control**	**ERAS**	**Control**	
Duzgun (28)	Retrospective	NR	62	40	41.9	42.5	57.3 ± 12.5	56.1 ± 12.2	12.8 ± 6.3	12.4 ± 6.1	Colorectum: 26 Ovary: 11 Sarcomatosis: 8 Gastric: 5 Other: 12	Colorectum: 20 Ovary: 8 Sarcomatosis: 6 Gastric: 5 Other: 11	7.9 ± 4.9 h	6.6 ± 2.7 h	5
Webb, et al. (26)	Retrospective	Cisplatin, Mitomycin	81	49	52	53	54.4 ± 13.4	56 ± 10.9	12 ± 8.4	11.5 ± 9.4	Appendix: 47 Colon: 18 Mesothelioma: 7 Ovarian: 2 Gastric: 5 Small bowel: 1 Other: 1	Appendix: 26 Colon: 14 Mesothelioma: 4 Ovarian: 3 Gastric: 0 Small bowel: 1 Other: 1	6.5 ± 2.7 h	6.5 ± 2.2 h	6
Siddharthan, et al. (19)	Retrospective	Mitomycin	15	16	NR	NR	60 (36–73)	57 (31–72)	3 (0–26)	6 (0–18)	NR	NR	418 (270–590) mins	452 (278–780) mins	5
Lu, et al. (18)	Retrospective	Mitomycin	20	11	35	36.4	50 (46–58)	47 (43–55)	13.5 (10–16.5)	10 (8–15)	Appendix: 12 Colorectal: 4 Other: 4	Appendix: 7 Colorectal: 4 Other: 0	347 (303.5–412.5) mins	391 (351–490) mins	5
Martin, et al. (27)	Retrospective	Oxaliplatin, Mitomycin	20	105	40	59	51.7 (34.5–71.2)	58.7 (25.1–80)	NR	NR	Appendix: 9 Colorectal: 8 Gastric: 1 Primary peritoneal: 1 Other: 1	Appendix: 35 Colorectal: 42 Gastric: 3 Primary peritoneal: 7 Other: 10	NR	NR	6
White, et al. (25)	Retrospective	Cisplatin, Mitomycin	80	88	37.5	36.4	56.5 ± 12.4	56.7 ± 12.2	13.2 ± 9.4	13.6 ± 8.9	NR	NR	370 ± 106 mins	360 ± 118 mins	6

*ERAS, enhanced recovery after surgery; PCI, peritoneal cancer index; Mins, minutes; h, hours; NR, not reported; NOS, Newcastle-Ottawa scale*.

**Table 2:** ERAS elements in the included studies.

**References**	**Pre-operative**	**Intra-operative**	**Post-operative**
Duzgun, et al. (28)	Preadmission education No bowel pre-paration Prophylaxis against VTE Nutrition Physiotherapy	Warming Thoracic epidural analgesia Goal-directed liquid management	Removed nasogastric tube and drains Early oral nutrition Early ambulation Use of chewing gum
Webb, et al. (26)	Routine protein and carbohydrate supplementation	Goal directed/balanced fluids	Clear liquid diet POD 0, advance as tolerated, no feeding tube
		Multimodal pain therapy, including TAP block	Use of drains and tubes only when indicated, Intermediate/Step-down
Siddharthan, et al. (19)	Pre-habilitation	Epidural Placement	Removal of nasogastric tube on post-operative day 1
	High protein diet for 30 days Carbohydrate loading	Goal directed fluid resuscitation Limit intravenous narcotics	Removal of Foley catheter on post-operative day 2
	Mechanical bowel pre-paration with antibiotics		Diet on post-operative day 1 Early mobilization Multimodal pain control
Lu, et al. (18)	Pre-admission education	Zero fluid balance/Goal directed fluid therapy	Fluids x 6 h only
	Pre-operative carbohydrate beverage	Urine output 0.25 cc/kg/h tolerated	Fluid bolus only for clinical concern
	Mechanical bowel pre-paration	Multimodal pain therapy, opioid sparing	Early mobilization
	No preoperative fluid bolus	TAP blocks	Pain control: Maximize oral non-narcotic medicines
	Gabapentin, celecoxib, acetaminophen, Prophylaxis against VTE, Antibiotic prophylaxis Epidural placement		Postop diet: POD0 clear fluids, POD 1 toast crackers, POD 2 regular diet
Martin, et al. (27)	Pre-operative education	Time hypothermic (defined as <35 C (<95 F)	Riddance of nasogastric tube
	Nutritional optimization Clear liquids before surgery	Intra-operative opioid-sparing Goal-directed fluid therapy	Post-operative nausea and vomiting management
	Appropriate bowel prep Pre-operative pain management education	Appropriate Foley catheter use	Early nutrition Early mobilization
White, et al. (25)	Pre-operative education	Goal-directed fluid management	Tylenol 1,000 mg every 6 h
	Pre-habitation if needed	500 mL 5% albumin	Early ambulation
	Nutritional support if needed	Crystalloid rate: 3 mL/kg/h Lidocaine infusion	PCA, early switch to oral pain medications
	Nothing by mouth 4 h before surgery	Sodium thiosulphate, manitol, magnesium with cisplatin	Removal of nasogastric tube and Foley on POD 1
	Tylenol 1,000 mg	No routine nasogastric tube use	Clear liquid diet POD 1

*VTE, venous thromboembolism; POD, postoperative day; TAP, transverse abdominis plane*.

**Figure 2:**
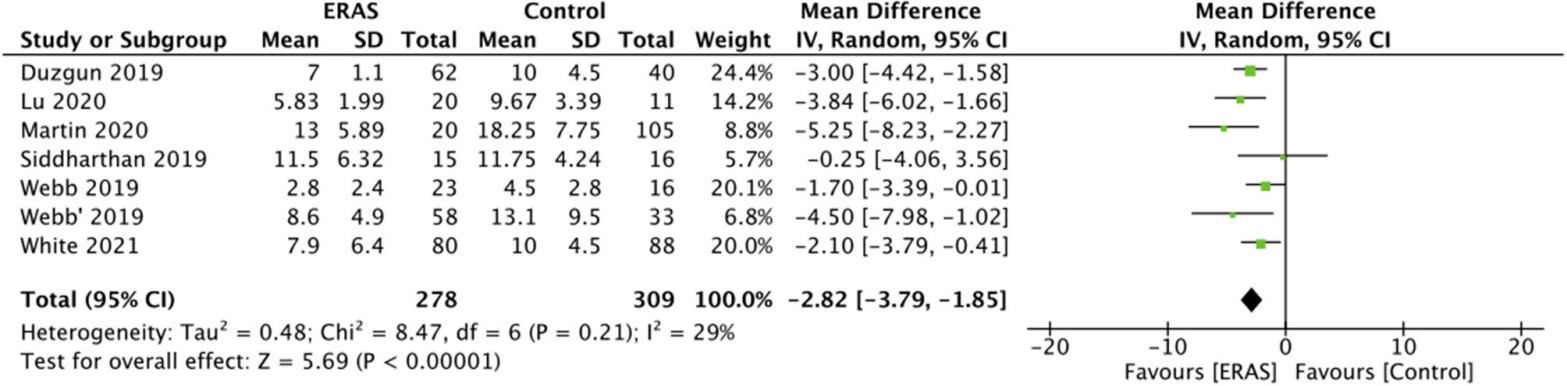
Meta-analysis for LOS between ERAS and control groups.

**Figure 3:**
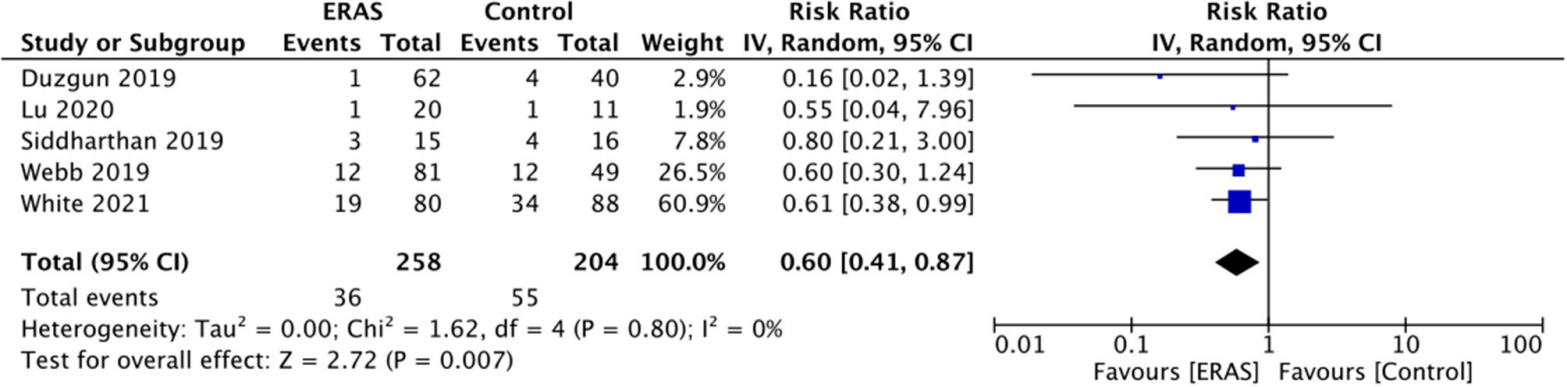
Meta-analysis for grade III/IV complications between ERAS and control groups.

LOS was reported by all included studies. A meta-analysis of data of 278 patients in the ERAS group and 309 patients in the control group indicated a statistically significant reduction in LOS with the ERAS protocol (MD: −2.82 95% CI: −3.79, −1.85 I^2^ 2 = 9% *p* < 0.00001) (Figure 2). There was no evidence of publication bias (Supplementary Figure 1). While data on complications were reported by all included studies, the majority (five studies) reported incidence of higher grade complications (Calvien Dindo grade III/IV) only. Hence, only such data could be pooled for the analysis. Our results demonstrated a significantly reduced risk of grade III/IV complications with the use of ERAS protocol as compared to the control group (RR: 0.60 95% CI: 0.41, 0.87 I^2^ 0 ^=^ % *p* = 0.007) (Figure 3). There was no evidence of publication bias (Supplementary Figure 2).

**Figure 4:**
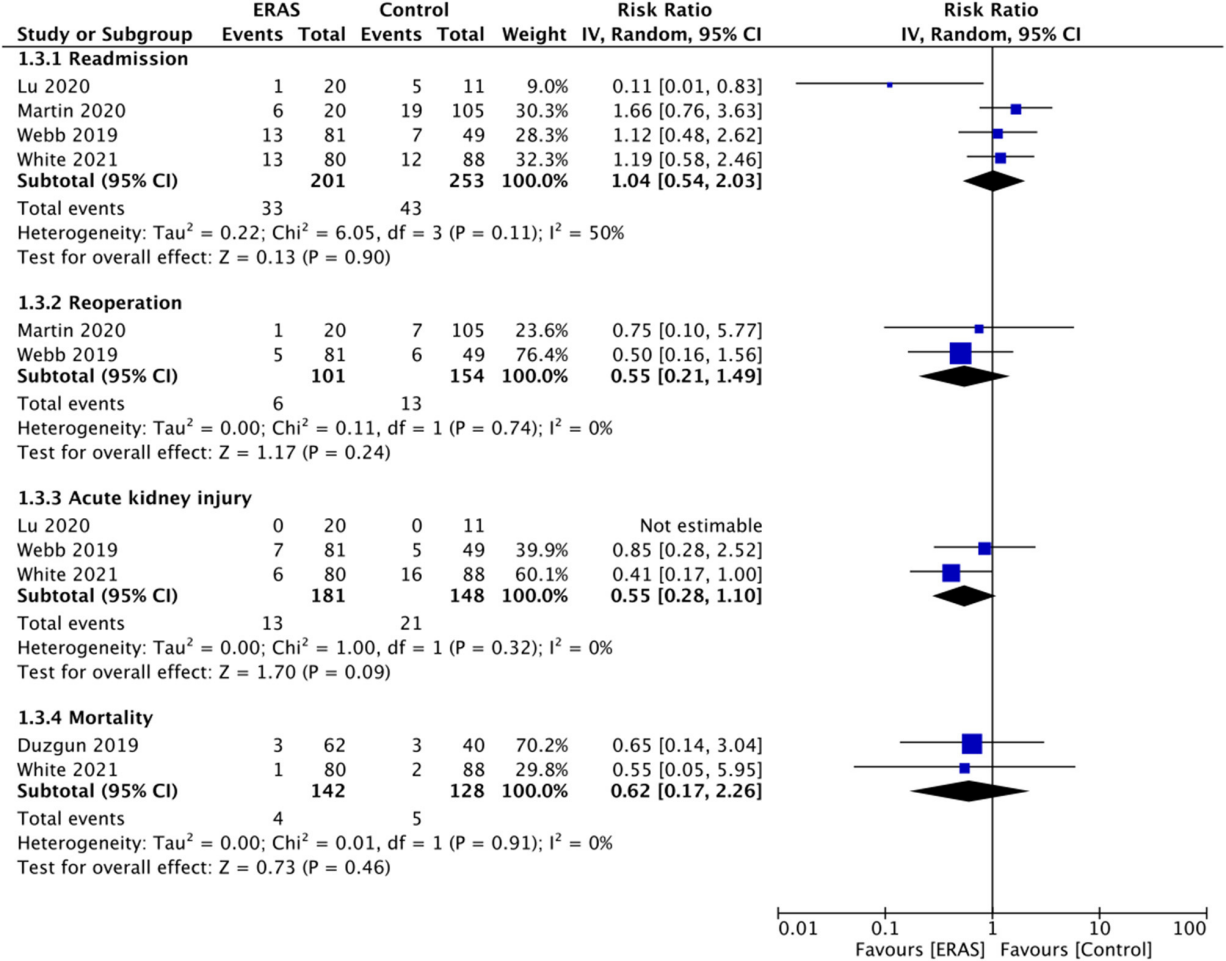
Meta-analysis for readmission, reoperation, AKI and mortality between ERAS and control groups.

Data on early reoperation and readmission rates were reported by four and two studies, respectively. Pooled analysis demonstrated no statistically significant difference in the risk of early reoperation (RR: 1.04 95% CI: 0.54, 2.03 I2 5 = 0% *p* = 0.90) or readmission (RR: 0.55 95% CI: 0.21, 1.49 I^2^ 0 ^=^ % *p* = 0.24) between the two study groups (Figure 4). Only three studies reported data on the incidence of postoperative AKI. Meta-analysis failed to demonstrate any statistically significant difference between the ERAS and control groups (RR: 0.55 95% CI: 0.28, 1.10 I2 0 = % *p* = 0.09) (Figure 4). Similarly, on a pooled analysis of data from just two studies, we did not find any statistically significant difference in the risk of mortality between the two groups (RR: 0.62 95% CI: 0.17, 2.26 I2 0 = % *p* = 0.46) (Figure 4).

## Discussion

The core principle of ERAS protocol is to standardized and optimize patient care to harmonize the exaggerated inflammatory surgical response which is associated with adverse patient outcomes (12). Specifically, the foundation of ERAS is built on several elements like patient education, nutritional screening, multimodal opioid-sparing anesthesia, controlled perioperative fluid management, early feeding and ambulation, early removal of catheters and drains, prevention of postoperative nausea and vomiting (PONV) along with other ancillary guidelines depending on the surgery type (13). Since its success with colorectal surgery, the program has been adopted in several surgical specialties with different guidelines according to the surgery type (29, 30). The ERAS society has also recently published recommendations to standardize the methodology of development of these guidelines (31).

Owing to the historically high rates of mortality and morbidity of CRS + HIPEC (7, 8), hesitation exists amongst clinicians to completely utilize the program for these patients despite reports of improved outcomes with singular elements of ERAS protocol. Hendrix et al. (16) in a sample size of 169 CRS + HIPEC patients have demonstrated that intraoperative restrictive fluid therapy with standard monitoring is associated with reduced LOS and grade III/IV complications. Similarly, Osseis et al. (17) have shown that clear preoperative education significantly reduced LOS, time to first ambulation, and patient satisfaction in CRS + HIPEC patients. Indeed, goal-directed intraoperative fluids as part of the ERAS program were used by all included studies in this review while patient education was utilized by five of the six studies. Important to note is that detailed ERAS guidelines specific to CRS + HIPEC have been made available only recently much after the conduct of the included studies (32, 33). Hubner et al. (32, 33) in August 2020 provided several preoperative, intraoperative, and postoperative recommendations for CRS + HIPEC, focusing on the core tenants of the ERAS program mentioned earlier. However, of the 72 items on the list, direct evidence was available only for eight items and the rest were extrapolated from other related colorectal and abdominal surgical procedures. Indeed, the paucity of evidence is a major limiting factor in the application of several elements of the ERAS program to CRS + HIPEC patients.

On a detailed analysis of the ERAS protocol, it can be noted that there was variation in the elements of the ERAS across the six included studies. This was expected owing to the different participating centers and timelines of the studies; and it is has been a factor of heterogeneity in other meta-analysis studies on ERAS programs as well (13, 34). Nevertheless, several core principles were utilized by majority studies like preoperative education, nutrition optimization, goal-directed fluid management, use of regional blocks or multimodal anesthesia, early removal of tubes and drains, and postoperative pain control. Indeed, our meta-analysis indicated that the application of ERAS protocol significantly reduced LOS for CRS + HIPEC patients. Individually, all of the included studies demonstrated a significantly reduced LOS, except for Siddharthan et al. (19). The lack of difference in this study could be attributed to its small sample size. Our results concur with other meta-analysis studies on the ERAS program. Tan et al. (35) on breast reconstruction, Malczak et al. (34) on bariatric surgery, Zhuang et al. (13) on colorectal surgery; have all demonstrated that implementation of ERAS leads to significantly reduced LOS.

According to Alyami et al. (36), the rate of major complications after CRS + HIPEC based on Calvien Dindo classification can be as high as 25%. In our analysis, the rates of grade III/IV complications in the control group were close to this figure at 26.9%. However, in the ERAS group, the incidence was 13.9% with a statistically significant 40% reduced risk of complications. Owing to the different ERAS elements used in the studies, it is difficult to delineate which intervention or interventions might have contributed to these results. CRS + HIPEC procedure is associated with large hemodynamic changes due to surgical, chemical, and hyperthermic trauma and inadequate volume resuscitation can lead to hemodynamic instability, hypoperfusion, and nephrotoxicity while excessive fluids can cause overload, tissue edema, and a higher risk of major complications (16). The use of goal-directed fluid therapy may therefore be an important contributor to the reduced incidence of complications (16). Furthermore, nutrition support and other preoperative measures like physiotherapy, adequate pre-anesthetic screening for comorbidities like diabetes can also contribute to a reduced risk of infectious complications with ERAS (28).

There have been concerns with the use of the ERAS program which include risk of readmission and AKI (14, 15). Increased early readmission rates with ERAS are, however, not universal with research also indicating a reduced risk of readmission with the program (37). Important contributors to early readmission with abdominal surgical procedures are postoperative ileus and infectious complications (27). However, Francis et al. (38) have demonstrated that poor compliance to ERAS elements is an independent predictor of early readmissions. While the majority of studies in our review did not report percentage compliance with the ERAS protocol, analysis of limited data revealed that application of ERAS does not increase early readmission, reoperation, or mortality in patients undergoing CRS + HIPEC. The scarce data also limited our ability to assess the rates of postoperative ileus and infectious complications.

The chemotherapeutic agents used with HIPEC especially cisplatin can lead to significant renal injury and there are apprehensions that restricted use of fluids in the perioperative period may exacerbate it (16, 39). However, studies assessing restrictive fluid therapy for CRS + HIPEC have failed to demonstrate such association (16, 40). In our meta-analysis, only limited studies reported the impact of ERAS on AKI. Although the results were non-significant, in view of the scarce data, it is difficult to comment on the actual association between ERAS and AKI in patients undergoing CRS + HIPEC. It is important to note that restriction of fluid therapy can be difficult in patient undergoing CRS + HIPEC. Fleres et al. (41) have demonstrated that cisplatin levels with HIPEC remain high up to the 4th post-operative day and return to preoperative levels only by the 7th post-operative day. The authors noted that hyperhydration along with infusion of colloids is of particular importance in the first four days after the procedure. In this context, application of ERAS can be difficult to apply in all patients undergoing CRS + HIPEC and may be restricted to patients with low peritoneal cancer index (PCI) undergoing minor resection.

The results of our study should be interpreted with the following limitations. Foremost, only six studies were available for review mostly with a small sample size. Furthermore, all were retrospective studies and prone to bias. Most importantly, none of the studies conducted baseline matching and this could have skewed our outcomes. The ERAS and control protocols were not parallelly followed in the included studies. The different periods of the study groups could have been associated with other changes in hospital practices which may have influenced outcomes. Secondly, data for all variables were not universally reported by the included studies. Some of the outcome variables were analyzed with just two or three studies which restricted the power of our analysis. We also could not analyze the impact of ERAS on several important variables like pain scores, post-operative ileus, hospitalization costs, etc. Thirdly, there was methodological heterogeneity across studies in the ERAS elements used. The compliance for these elements was not known in most studies and this may have impacted outcomes.

Nevertheless, our study is the first systematic review and meta-analysis comparing ERAS with no-ERAS protocols for patients undergoing CRS + HIPEC. The pooled analysis of six studies overcomes the limitation of a small sample size of individual studies and presents the largest dataset on the impact of ERAS on CRS + HIPEC.

For patients undergoing CRS + HIPEC, our results indicate that ERAS is associated with significantly reduced LOS along with lower incidence of complications. Limited data suggest that use of ERAS protocol is not associated with increased readmission, reoperation, and mortality rates in these patients. There is a need for RCTs to corroborate the current evidence. Future studies should focus on the incidence of AKI with ERAS in patients undergoing CRS + HIPEC. Trials should also be conducted on a highly selected group of patients with low PCI and minor resections in order to segregate the efficacy of ERAS in this cohort.

## Data Availability Statement

The original contributions presented in the study are included in the article/Supplementary Material, further inquiries can be directed to the corresponding author/s.

## Author Contributions

FM designed the study. Both the authors were involved in data acquisition, analysis, and synthesis. Both authors wrote, edited and approved the manuscript.

## Conflict of Interest

The authors declare that the research was conducted in the absence of any commercial or financial relationships that could be construed as a potential conflict of interest.

## Supplementary Material

Supplementary Figure 1 Funnel plot for the meta-analysis of LOS.

Supplementary Figure 2 Funnel plot for the meta-analysis of grade III/IV complications.
