# Supplementary figures and images for: Enhanced Recovery After Surgery for Patients Undergoing Cytoreductive Surgery and Hyperthermic Intraperitoneal Chemotherapy: A Systematic Review and Meta-Analysis
Source: Front Surg. 2021 Jul 21;8:713171. doi: 10.3389/fsurg.2021.713171 (PMC8336690; doi:10.3389/fsurg.2021.713171)

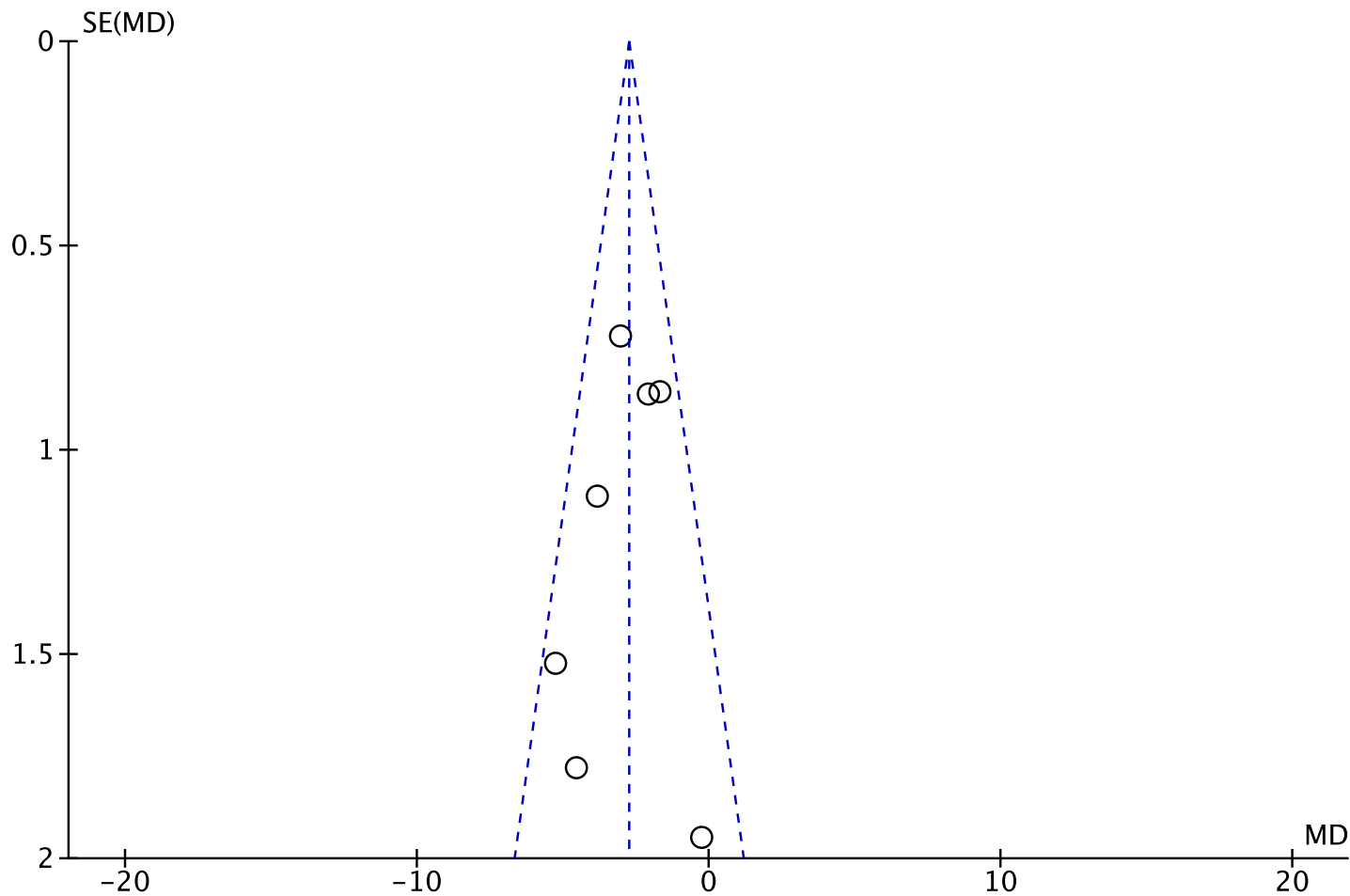

Supplement: Supplementary Figure 1 — Funnel plot for the meta-analysis of LOS. [file Data_Sheet_1.PDF]

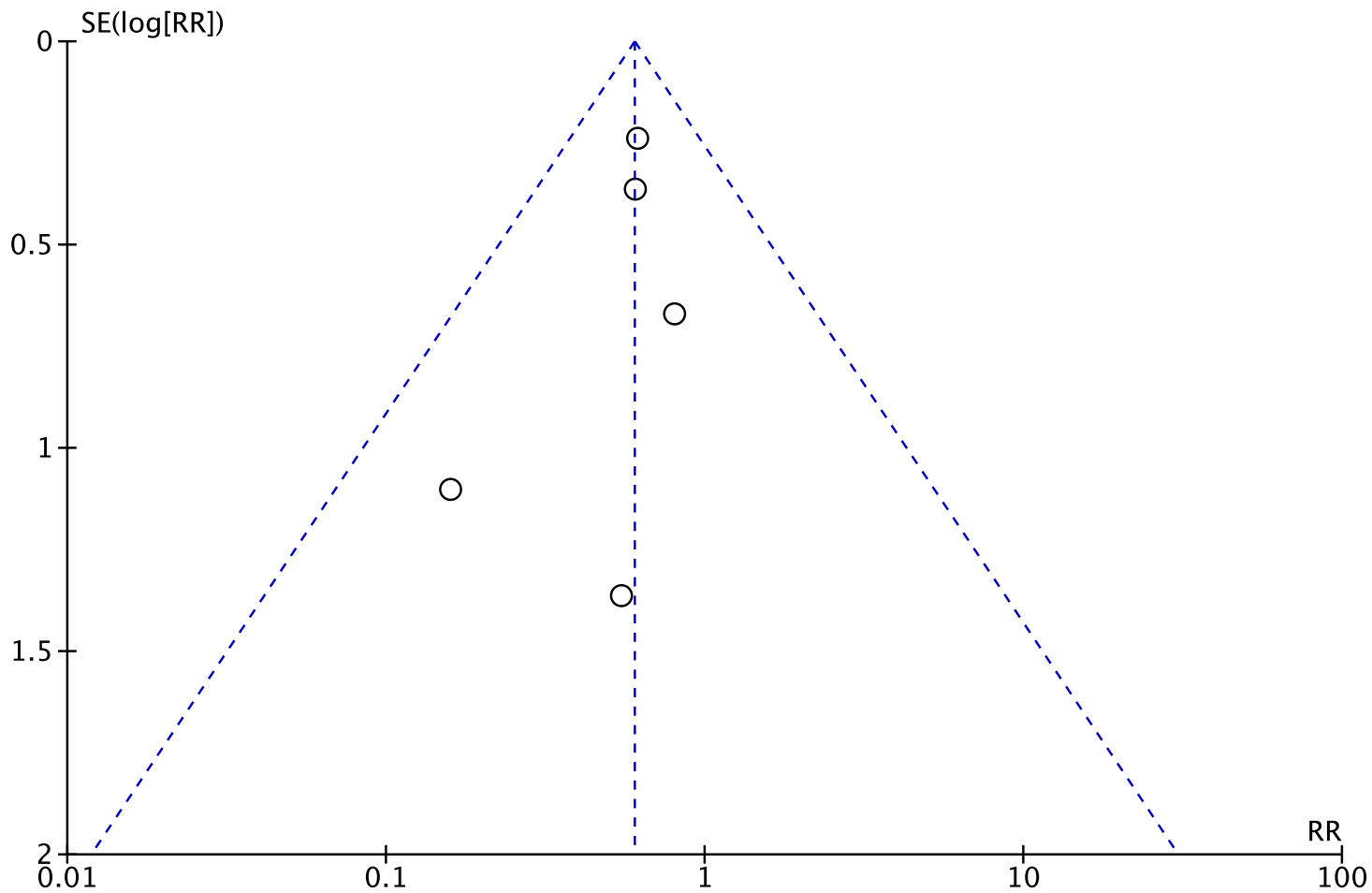

Supplement: Supplementary Figure 2 — Funnel plot for the meta-analysis of grade III/IV complications. [file Data_Sheet_2.PDF]
