# Supplementary material for: Enhanced Recovery After Surgery for Patients Undergoing Cytoreductive Surgery and Hyperthermic Intraperitoneal Chemotherapy: A Systematic Review and Meta-Analysis
Source: Front Surg. 2021 Jul 21;8:713171. doi: 10.3389/fsurg.2021.713171 (PMC8336690; doi:10.3389/fsurg.2021.713171)
Supplement: Supplementary file 3 [file Table_1.DOCX]

Table 1: Search strategy and results of PubMed database

| **Search number** | **Query** | **Search Details** | **Results** |
| --- | --- | --- | --- |
| **1** | (enhanced recovery) AND (cytoreductive surgery) | ("enhance"[All Fields] OR "enhanced"[All Fields] OR "enhancement"[All Fields] OR "enhancements"[All Fields] OR "enhancer"[All Fields] OR "enhancer s"[All Fields] OR "enhancers"[All Fields] OR "enhances"[All Fields] OR "enhancing"[All Fields]) AND ("recoveries"[All Fields] OR "recovery"[All Fields]) AND ("cytoreduction surgical procedures"[MeSH Terms] OR ("cytoreduction"[All Fields] AND "surgical"[All Fields] AND "procedures"[All Fields]) OR "cytoreduction surgical procedures"[All Fields] OR ("cytoreductive"[All Fields] AND "surgery"[All Fields]) OR "cytoreductive surgery"[All Fields]) | 28 |
| **2** | (fast recovery) AND (cytoreductive surgery) | ("fasting"[MeSH Terms] OR "fasting"[All Fields] OR "fast"[All Fields]) AND ("recoveries"[All Fields] OR "recovery"[All Fields]) AND ("cytoreduction surgical procedures"[MeSH Terms] OR ("cytoreduction"[All Fields] AND "surgical"[All Fields] AND "procedures"[All Fields]) OR "cytoreduction surgical procedures"[All Fields] OR ("cytoreductive"[All Fields] AND "surgery"[All Fields]) OR "cytoreductive surgery"[All Fields]) | 6 |
| **3** | (accelerated rehabilitation) AND (cytoreductive surgery) - Schema: all | "accelerated"[All Fields] AND "rehabilitation"[All Fields] AND ("cytoreductive"[All Fields] AND "surgery"[All Fields]) | 0 |
| **4** | (multimodal perioperative care) AND (cytoreductive surgery) | ("multimodal imaging"[MeSH Terms] OR ("multimodal"[All Fields] AND "imaging"[All Fields]) OR "multimodal imaging"[All Fields] OR "multimodality"[All Fields] OR "multimodal"[All Fields] OR "multimodalities"[All Fields]) AND ("perioperative care"[MeSH Terms] OR ("perioperative"[All Fields] AND "care"[All Fields]) OR "perioperative care"[All Fields]) AND ("cytoreduction surgical procedures"[MeSH Terms] OR ("cytoreduction"[All Fields] AND "surgical"[All Fields] AND "procedures"[All Fields]) OR "cytoreduction surgical procedures"[All Fields] OR ("cytoreductive"[All Fields] AND "surgery"[All Fields]) OR "cytoreductive surgery"[All Fields]) | 28 |
| **5** | (enhanced recovery) AND (hyperthermic intraperitoneal chemotherapy) | ("enhance"[All Fields] OR "enhanced"[All Fields] OR "enhancement"[All Fields] OR "enhancements"[All Fields] OR "enhancer"[All Fields] OR "enhancer s"[All Fields] OR "enhancers"[All Fields] OR "enhances"[All Fields] OR "enhancing"[All Fields]) AND ("recoveries"[All Fields] OR "recovery"[All Fields]) AND ("hyperthermic intraperitoneal chemotherapy"[MeSH Terms] OR ("hyperthermic"[All Fields] AND "intraperitoneal"[All Fields] AND "chemotherapy"[All Fields]) OR "hyperthermic intraperitoneal chemotherapy"[All Fields]) | 20 |
| **6** | (fast recovery) AND (hyperthermic intraperitoneal chemotherapy) | ("fasting"[MeSH Terms] OR "fasting"[All Fields] OR "fast"[All Fields]) AND ("recoveries"[All Fields] OR "recovery"[All Fields]) AND ("hyperthermic intraperitoneal chemotherapy"[MeSH Terms] OR ("hyperthermic"[All Fields] AND "intraperitoneal"[All Fields] AND "chemotherapy"[All Fields]) OR "hyperthermic intraperitoneal chemotherapy"[All Fields]) | 7 |
| **7** | (accelerated rehabilitation) AND (hyperthermic intraperitoneal chemotherapy) | "accelerated"[All Fields] AND "rehabilitation"[All Fields] AND ("hyperthermic"[All Fields] AND "intraperitoneal"[All Fields] AND "chemotherapy"[All Fields]) | 0 |
| **8** | (multimodal perioperative care) AND (hyperthermic intraperitoneal chemotherapy) | ("multimodal imaging"[MeSH Terms] OR ("multimodal"[All Fields] AND "imaging"[All Fields]) OR "multimodal imaging"[All Fields] OR "multimodality"[All Fields] OR "multimodal"[All Fields] OR "multimodalities"[All Fields]) AND ("perioperative care"[MeSH Terms] OR ("perioperative"[All Fields] AND "care"[All Fields]) OR "perioperative care"[All Fields]) AND ("hyperthermic intraperitoneal chemotherapy"[MeSH Terms] OR ("hyperthermic"[All Fields] AND "intraperitoneal"[All Fields] AND "chemotherapy"[All Fields]) OR "hyperthermic intraperitoneal chemotherapy"[All Fields]) | 14 |
| **9** | (ERAS) AND (cytoreductive surgery) | "ERAS"[All Fields] AND ("cytoreduction surgical procedures"[MeSH Terms] OR ("cytoreduction"[All Fields] AND "surgical"[All Fields] AND "procedures"[All Fields]) OR "cytoreduction surgical procedures"[All Fields] OR ("cytoreductive"[All Fields] AND "surgery"[All Fields]) OR "cytoreductive surgery"[All Fields]) | 23 |
| **10** | (ERAS) AND (hyperthermic intraperitoneal chemotherapy) | "ERAS"[All Fields] AND ("hyperthermic intraperitoneal chemotherapy"[MeSH Terms] OR ("hyperthermic"[All Fields] AND "intraperitoneal"[All Fields] AND "chemotherapy"[All Fields]) OR "hyperthermic intraperitoneal chemotherapy"[All Fields]) | 11 |
| **11** | (fast-track surgery) AND (Cytoreductive Surgery) | "fast-track"[All Fields] AND ("surgery"[MeSH Subheading] OR "surgery"[All Fields] OR "surgical procedures, operative"[MeSH Terms] OR ("surgical"[All Fields] AND "procedures"[All Fields] AND "operative"[All Fields]) OR "operative surgical procedures"[All Fields] OR "general surgery"[MeSH Terms] OR ("general"[All Fields] AND "surgery"[All Fields]) OR "general surgery"[All Fields] OR "surgery s"[All Fields] OR "surgerys"[All Fields] OR "surgeries"[All Fields]) AND ("cytoreduction surgical procedures"[MeSH Terms] OR ("cytoreduction"[All Fields] AND "surgical"[All Fields] AND "procedures"[All Fields]) OR "cytoreduction surgical procedures"[All Fields] OR ("cytoreductive"[All Fields] AND "surgery"[All Fields]) OR "cytoreductive surgery"[All Fields]) | 6 |
| **12** | (fast-track surgery) AND (Hyperthermic Intraperitoneal Chemotherapy) | "fast-track"[All Fields] AND ("surgery"[MeSH Subheading] OR "surgery"[All Fields] OR "surgical procedures, operative"[MeSH Terms] OR ("surgical"[All Fields] AND "procedures"[All Fields] AND "operative"[All Fields]) OR "operative surgical procedures"[All Fields] OR "general surgery"[MeSH Terms] OR ("general"[All Fields] AND "surgery"[All Fields]) OR "general surgery"[All Fields] OR "surgery s"[All Fields] OR "surgerys"[All Fields] OR "surgeries"[All Fields]) AND ("hyperthermic intraperitoneal chemotherapy"[MeSH Terms] OR ("hyperthermic"[All Fields] AND "intraperitoneal"[All Fields] AND "chemotherapy"[All Fields]) OR "hyperthermic intraperitoneal chemotherapy"[All Fields]) | 6 |
